# Stakeholder Perspectives on a Community-Based Peer-Led Biomedical HIV Prevention Intervention for Women Who Engage in Sex Work in Southwestern Uganda: Results from a Qualitative Study

**DOI:** 10.1007/s10461-025-04782-z

**Published:** 2025-06-09

**Authors:** Cecilia Akatukwasa, Jennifer Velloza, Milliam Korukiiko, Richard Aruho, Laura B. Balzer, James F. Rooney, Moses R. Kamya, Catherine A. Koss, Jane Kabami

**Affiliations:** 1 Infectious Diseases Research Collaboration, Kampala, Uganda; 2 Department of Epidemiology & Biostatistics, University of California San Francisco, 550 16th Street 2nd Floor, San Francisco, CA 94158 USA; 3 Division of Biostatistics, School of Public Health, University of California Berkeley, Berkeley, CA USA; 4 Gilead Sciences, Foster City, CA USA; 5 Division of HIV, Infectious Diseases, and Global Medicine, University of California San Francisco, San Francisco, CA USA; 6 School of Medicine, College of Health Sciences, Makerere University, Kampala, Uganda

**Keywords:** PrEP, PEP, HIV, Uganda, Peer delivery

## Abstract

HIV prevalence among Ugandan women who engage in sex work (WESW) is 31%, yet uptake of oral pre-exposure prophylaxis (PrEP) and post-exposure prophylaxis (PEP) is suboptimal due to multiple factors, including stigma and barriers to accessing healthcare. “Peer mentors” (peer leaders within WESW networks) could facilitate community-based delivery of biomedical HIV prevention products for WESW. We conducted formative research with key stakeholders to refine a potential community-based, peer mentor-led PrEP/PEP intervention. From July-August 2023, we conducted focus group discussions (FGDs) and key informant interviews (KIIs), informed by the Capability, Opportunity, Motivation and Behavior (COM-B) model. Interview guides explored determinants of PrEP/PEP use, acceptability of hypothesized intervention components (monthly PrEP/PEP counseling, HIV self-testing [HIVST], peer delivery of oral PrEP refills, phone/SMS hotline for peer support, rapid PEP access), and elicited ideas about additional intervention components. We recruited WESW, peer mentors, providers, and implementing partners in southwestern Uganda. FGD and KII transcripts were analyzed using a rapid qualitative analysis approach. We conducted four FGDs with WESW (*N* = 20) and peer mentors (*N* = 21) and nine KIIs with providers (*N* = 4) and implementing partners (*N* = 5). Most described substantial interest in a peer-led oral PrEP/PEP model for WESW. Community-based PrEP/PEP delivery with flexible hours and locations (e.g., bars, lodges) was suggested to address barriers to accessing health facilities. Peer mentors were perceived as trusted agents to increase PrEP/PEP awareness and deliver person-centered care for WESW, in partnership with clinicians. Participants emphasized the need for comprehensive and ongoing peer mentor training and supervision. Integrated PrEP/PEP and HIVST provision were also described as key intervention components to empower WESW and support differentiated service delivery. A peer-led oral PrEP/PEP delivery strategy could address key barriers to biomedical HIV prevention use among WESW in Uganda. Subsequent research is needed to test the impact of this approach on PrEP/PEP use among WESW.

## Introduction

Women who engage in sex work (WESW) play a critical role in the dynamics of the HIV epidemic in eastern and southern Africa [1]. In 2022, the risk of acquiring HIV was nine times higher for WESW than among in the wider adult (aged 15–49 years) population globally [2]. From 2010 to 2022, WESW contributed approximately 8% of all new HIV diagnoses globally and, in Uganda, 31% of the estimated 130,000 WESW are living with HIV [2–4].

Pre-exposure prophylaxis (PrEP) and post-exposure prophylaxis (PEP) are highly effective HIV prevention interventions [5–7], which could be powerful, female-controlled HIV prevention tools for WESW. WESW may experience inconsistent condom use due to power imbalances, client preferences, or economic pressures to accept higher payments for condomless sex [8]. Daily oral PrEP with tenofovir disoproxil fumarate and emtricitabine or lamivudine is recommended by the Ministry of Health and available through public facilities in Uganda [9, 10]. WESW have reported high interest and initial uptake, but retention through 12 months is low [11–13]. For example, a study conducted in Kampala reported that about half of WESW continued on PrEP through six months [14]. PEP (an oral pill taken daily for 28 days after HIV exposure) is primarily reserved for health care exposures and gender-based violence. However, use as a primary HIV prevention strategy has been limited.

There are multiple barriers to the utilization of oral PrEP and PEP among WESW globally. Structural factors, such as stigma and provider attitudes can deter WESW from seeking HIV prevention services [15, 16]. Additionally, the legal status of sex work in many countries forces WESW to operate in clandestine environments, limiting healthcare access [17]. Economic constraints also play a role, with many WESW prioritizing their immediate income needs over preventive health interventions [18]. Furthermore, a lack of awareness and misconceptions about PrEP have been reported as major barriers to uptake and retention [19]. There is a pressing need for innovative strategies that can address these multi-level barriers to HIV prevention services for WESW in Uganda.

Peer-led interventions have emerged as a promising approach to HIV prevention in some populations, including WESW [20]. By involving peers, interventions can increase social support, trust, and engagement, and ensure delivery of culturally-relevant services in community settings. Leveraging the trust and shared experiences within sex worker communities, peers can play a critical role in bridging the gap between WESW and healthcare services [21]. Studies have shown that peer-led interventions increase awareness and reduce stigma around HIV prevention, making oral PrEP and PEP more acceptable and accessible [22]. In contexts where stigma is prevalent, peers are often seen as more approachable than healthcare providers, enhancing trust in HIV prevention efforts [23]. By empowering WESW through peer engagement, these approaches can be instrumental in reducing HIV acquisition and improving health outcomes within this population. This study explored WESW, peer mentors, and other stakeholders’ perspectives of facilitators and barriers to PrEP and PEP delivery to inform a novel community-based, peer-led, biomedical HIV prevention intervention for WESW in southwestern Uganda.

## Methods

### Study Design

We conducted a formative, qualitative study to inform intervention development for a subsequent trial to evaluate a community-based oral PrEP and PEP peer delivery model for WESW in rural southwestern Uganda (NCT#06353295). This study was conducted in the context of government approval and momentum around peer-led HIV service delivery for WESW in Uganda [24, 25]. Currently, peer mentors may provide HIV education, demand creation for HIV prevention, and refer WESW to HIV testing and prevention services (such as condoms and PrEP) at health facilities or WESW-friendly drop-in-centers (DICs). Peer mentors may also participate in the distribution of HIV self-test kits, lubricants, and condoms.

In Uganda, standard follow-up for PrEP includes quarterly clinician visits for HIV testing and refills (with a 30-day supply at initiation) and peer mentors interact with clients as needed. Depending on PrEP client need, PrEP adherence after initiation, and availability of daily oral PrEP, quarterly oral PrEP refills may be provided after the second refill in accordance with differentiated service delivery guidance. Oral PrEP refills may also be offered at community locations in Uganda (as part of HIV testing and status-neutral medication delivery), depending on peer mentor availability and need in a given community. PEP is available at accredited facilities but peer mentors do not typically play a role in PEP delivery. We aimed to explore how the role of peer mentors could be expanded to facilitate oral PrEP and PEP delivery in community settings.

The study was conducted in two peri-urban communities in southwestern Uganda, Rubaare and Rwashamaire, in the Ntungamo district. These communities are transit hubs to larger cities and have a high concentration of venues where sex work may occur. Following stakeholder engagement meetings, we conducted focus group discussions (FGDs) with WESW and peer mentors and key informant interviews (KIIs) with health care providers and implementing partners to explore perspectives on PrEP and PEP use among WESW and gather insights on components of a peer-led oral PrEP and PEP delivery model to address any barriers to use for WESW in southwestern Uganda.

### Theoretical Model

This study was grounded in the the COM-B theoretical model (Capability, Opportunity, Motivation, and Behavior) [26]. The COM-B is focused on identifying client, provider, and clinic determinants of behavior and offers guidance on evidence-based intervention components to address each determinant and bring about behavior change for a target health behavior (e.g., effective oral PrEP and PEP use for WESW in Uganda). In FGDs with WESW, our target behavior of focus was oral PrEP and PEP use from the end-user perspective. In FGDs with peer mentors and KIIs, we sought to understand capability, opportunity, and motivation domains related to a target behavior of offering PrEP/PEP to WESW from the provider, implementer, or health system side. This dual focus allowed us to gather qualitative data on both demand- and supply-side facilitators and barriers to oral PrEP and PEP use and delivery for WESW. The COM-B model facilitated a detailed exploration of factors that drive oral PrEP and PEP access, uptake, and use among WESW which helped identify components of a peer-led PrEP/PEP intervention to target these factors.

### Study Participants

We conducted separate FGDs with purposive samples of WESW and peer mentors (defined as women who currently or previously engaged in sex work and provided HIV-related services to peers). We chose FGDs as the data collection modality for these groups because we felt that a group-based approach would help women open up, support feelings of social cohesion, and potentially help elicit conversations about stigma around PrEP and PEP use through different experiences. WESW and peer mentors were recruited for FGDs in collaboration with key stakeholders in each community, including community-based organizations and focal persons for the WESW community. WESW and peer mentors purposively selected from the Rubaare and Rwashamaire communities to ensure equal representation.

We held two FGDs with WESW. Eligible WESW were aged 18 years and over or mature minors aged 15 years and over, female sex at birth, and self-identified as commercial sex workers or exchanged sex for goods or money in the last 3 months. We held two FGDs with peer mentors. Eligible peer mentors included WESW who were actively engaged in providing support to other WESWs (as described above), and worked in settings associated with sex work.

We conducted KIIs with healthcare providers and implementing partners. Eligible providers included clinicians, nurses, counselors working in health facilities or DICs providing HIV services in Rubaare and Rwashamaire. We included implementing partners in HIV prevention programming and those who oversee health systems and health facilities (e.g., district health officers).

### Data Collection

A two-person research team MK, CA collected data from July-August 2023. FGDs included discussion of shared experiences related to concerns about HIV exposure (e.g., “Among co-workers/friends, how much concern do people have about their risk of getting infected with HIV?”), knowledge and prior use of oral PrEP and PEP (e.g., “What have you been told about PrEP?”), interest in a peer-led HIV prevention delivery (e.g., “What do you think we need to know about helping WESW access PrEP and PEP?”), support needed for peer HIV prevention service delivery (asked among peer mentors only; e.g., “What additional training would you need to deliver PrEP/PEP to WESW?”), and possible components of a peer-led, community-based oral PrEP and PEP delivery model. Each FGD included about 10 participants and was conducted by a trained facilitator using using semi-structured guides. A note-taker was present during each discussion. All FGDs were conducted in Runyankore and audio-recorded.

KIIs with health care providers and implementing partners explored faciliators and barriers to a peer-led intervention for oral PrEP/PEP delivery to WESW (e.g., “What will be challenges with maintaining this type of HIV prevention peer mentor approach?”) and perceptions of this approach (e.g., “What do you think will be the benefits of having peer mentors support PrEP and PEP delivery for WESW?”). KIIs were audio-recorded.

### Data Analysis

All audio-recordings were transcribed and translated into English. Qualitative data analysis was conducted by 5 study team members CA, JV, MK, CK, JK using a rapid data analysis approach (RDA) [27]. RDA is an intensive, team-based approach that is specifically important for time-sensitive projects, including those seeking to use qualitative data to inform intervention development [27]. Our RDA process included five steps. First, CA, JV, MK, CK and JK familiarized themselves with the data through readings of transcripts, noting key concepts relating to the research questions. Second, JV created domain names and summary templates based on a priori themes derived from the guides. Separate summary templates were developed for each FGD and IDI guide. Third, MK, CA, and JV piloted the templates, whereby each person separately completed summary templates for each of the same 2 FGD and 1 KII transcripts, to assess its usability and relevance the research questions. During this piloting step, MK, CA, and JV met to discuss summary templates and resolved any discrepancies by developing template instructions and modifying templates as needed. Fourth, CA, MK, and JV separately completed summary templates for the remaining transcripts. Fifth, JV transferred all points from summary templates into a data visualization matrix whereby each row represented a participant KII or FGD and each column represented themes for a particular domain of the summary template. As with the summary templates, separate matrices were developed for each guide. Reviewing data down columns within each matrix helped to identify key themes related to oral PrEP and PEP product use and opinions on a peer mentor-led PrEP/PEP delivery model. Reviewing data across the separate matrices revealed similarities and differences in perspectives on a peer-led PrEP/PEP delivery model across participant groups. We also used color-coding to signify themes around each of the COM-B model constructs (e.g., blue to highlight themes related to capability). JV, CA, MK, JK, and CK met regularly throughout the analysis process and JK and CK provided insights into emerging themes.

## Results

We conducted two FGDs with WESW (*N* = 20 total, 10 and 11 per FGD) with a median age of 25 years (range: 18–34), and two FGDs with peer mentors (*N* = 18 total, 10 and 8 per FGD) with a median age of 23 years (range: 19–35). We conducted IDIs with four HIV care providers (all women, including 3 nurses and 1 clinical officer), 2 PrEP and PEP implementing partners in Uganda, and 3 district/facility-level managers.

**Fig. 1:**
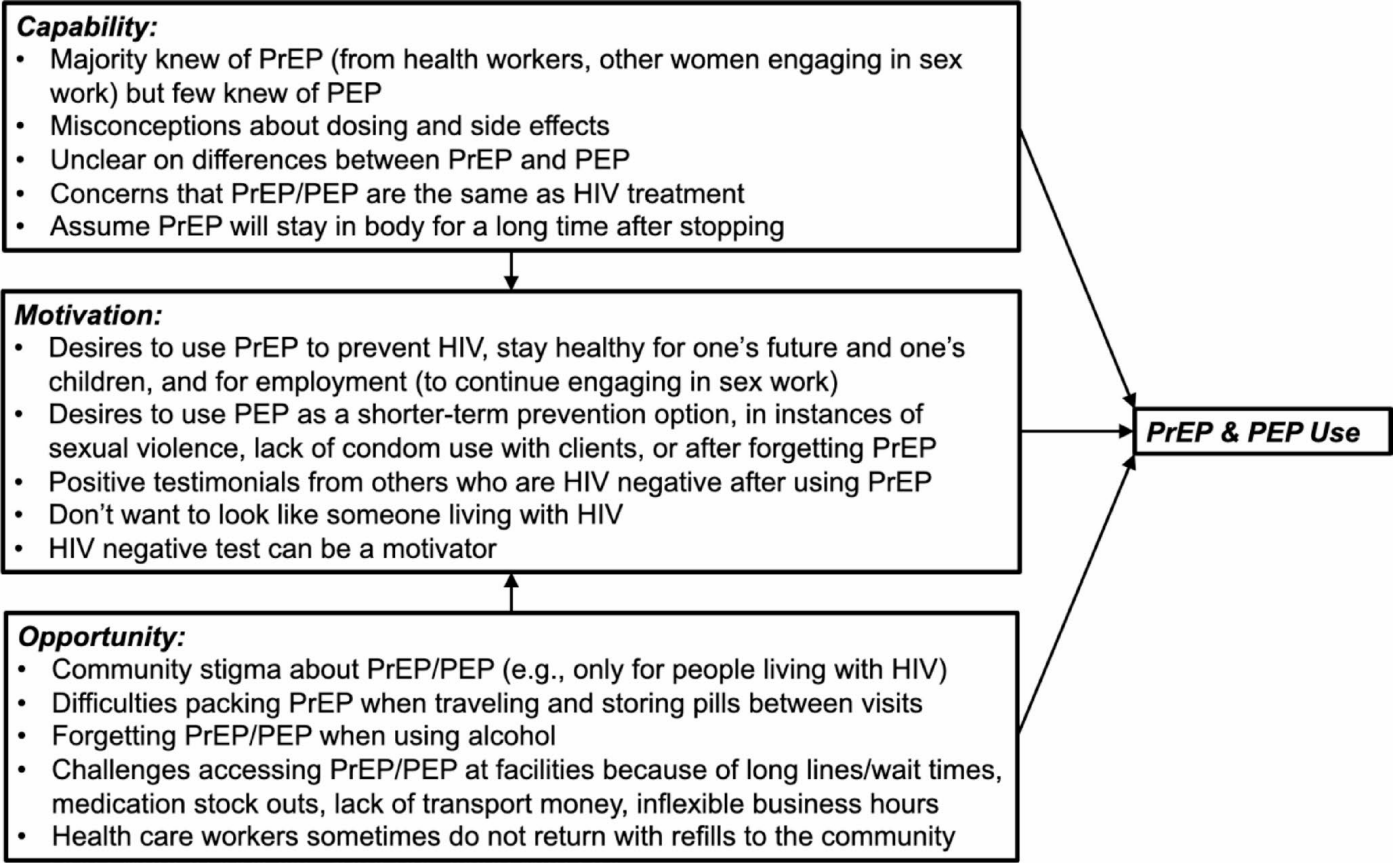
Key qualitative themes on barriers to PrEP and PEP use among women who engage in sex work, by COM-B construct. PrEP=pre−exposure prophylaxis; PEP=post−exposure prophylaxis; COM−B=Capability−Opportunity−Motivation Behavior Change Model

WESW described barriers and facilitators to PrEP and PEP access and adherence related to their “Capability”, “Opportunity”, and “Motivation” to engage in adherence behaviors (COM-B model constructs, Fig. 1). Discussions of capability and opportunity around PrEP and PEP use largely focused on barriers, while conversations about motivations were driven by themes around facilitators. Across all FGDs and IDIs, themes emerged related to a package of peer mentor-led strategies matched to address these barriers across these three COM-B constructs (Table 1). Themes are presented here by COM-B construct.

**Table 1:** Key qualitative themes on a peer-led strategies to address barriers to PrEP/PEP use among women who engage in sex work, informed by the COM-B

COM-B construct	Barriers to PrEP/PEP use among WESW	Proposed peer mentor-led strategies to address barriers to PrEP/PEP	Representative Quotes
Capability	• Misconceptions about PrEP and PEP (e.g., PrEP is the same as HIV treatment) • Limited knowledge of PEP • Limited knowledge of how PrEP and PEP differ • Concerns about side effects • Confusion about how long protection benefit is sustained after stopping	• Community-based PrEP demand creation from peer mentors • PrEP/PEP counseling from trained peer mentors who can speak from own experience • Relationship building between peer mentors and health facility staff to troubleshoot challenges and learn about PrEP/PEP • Peers’ ongoing support and reminders to address concerns that may arise	**PrEP provider on peer mentors as PrEP champions**: *“Peer mentors of course are like our PrEP champions. They are the ones who teach the community about PrEP and they bring fellow sex workers for the services.”* **Implementing partner on peer mentors to provide HIV counseling while discussing their own experiences**: *“Counseling is about information sharing. For them (peer mentors) we shall not ask them may be medical counseling*,* but we shall ask them to tell people about their lived experiences*,* so they already know what they do. All we need to give them is the skill of communication.”*
Opportunity	• Community stigma about PrEP • Inability to discreetly travel with PrEP • Alcohol use could lead to missed doses • Long lines at healthcare facilities • PrEP/PEP stock-outs • No money for transport to get to facility • Lack of safe places to store PrEP/PEP • Refills irregularly and infrequently offered at hotspots	• Peer delivery of PrEP/PEP at convenient community locations (e.g., bars, lodges, hotels, brothels, peer mentor’s home) • Phone/SMS hotline for WESW to request refills and report PrEP/PEP challenges • Flexible PrEP refill options (1-month or 3-month refills) depending on ability to store pill containers and adherence • PrEP/PEP counseling by peers, with potential for additional community or phone sessions as needed, to troubleshoot concerns	**Peer mentor on importance of community-based PrEP/PEP delivery**: *“ensure PrEP and PEP services reach the hotspot and the sex workers are aware of the services and can easily access them*,* and ensure that the [peer mentor] is facilitated to deliver to them at their hotspots (community areas where WESW are based).… majority of the sex workers don’t want to come to health facility to get their refills because of stigma that other people will think that they are HIV positive.”* **Provider on WESW preferring PrEP from a peer in the community**: *“I believe peer mentors are going to work better [for] PrEP uptake and also maybe with accessibility. If I knew my peer mentor who is giving me PrEP– that I am going to find her at hotel X or bar X – I may go there at any time I feel like*,* [rather] than waiting for the schedule of the facility.”*
Motivation	• PrEP/PEP use needs to align with one’s own desires to prevent HIV • Differing preferences around prevention options taken daily (PrEP) versus over a shorter time (PEP) • Differing prevention needs for preventing HIV with romantic partners, clients, and in instances of sexual violence or rape • PrEP/PEP disclosure can prevent business • Motivation depends on saliency of HIV (e.g., recent HIV test result)	• HIVST kits to empower WESW to know their HIV status and motivate PrEP/PEP use • Integrated service delivery to address multiple health needs among WESW • Rigorous peer mentor selection process by fellow WESW to ensure that peer mentors are trusted and respected community members	**WESW on value of hearing about PrEP/PEP from a peer with similar experiences and HIV prevention motivations**: *“A peer knows what it means*,* when she is told that you have been exposed to HIV and when you ask her to deliver like PEP. She may ‘put on your shoes’ and bring [PrEP/PEP] to you immediately because she would have experienced the same at some point and she can easily help…she will be there for us in case of any need for PEP or PrEP services.”* **WESW on HIVST to facilitate PrEP use**: *“There are female sex workers who have never tested for HIV and do not know about their HIV status*,* and they may prefer that HIV self-testing kit because no other person will access their test results and when they test negative they will also be in position to start taking PrEP.”*

^PrEP=pre−exposure prophylaxis; PEP=post−exposure prophylaxis; WESW=women who engage in sex work; COM−B=Capability−Opportunity−Motivation Behavior Change Model; HIVST=HIV self−test^

### Capability: Peer Mentors to Address Misconceptions and Concerns About Biomedical HIV Prevention

Knowledge (“psychological capability”) was a critical determinant of oral PrEP and PEP use. Most WESW had heard about daily oral PrEP previously, from health care workers at the local clinics, providers conducting outreach activities, and other WESW. In both FGDs, they described misconceptions about oral PrEP dosing (e.g., whether to dose daily during periods of risk) and side effects (e.g., whether it could cause weight gain). WESW also expressed confusion about what would happen if they stopped taking PrEP: *“When I am taking PrEP and then stop*,* can there be any side effects?”* (**WESW FGD #2**,** Participant #7**).

While daily oral PrEP was generally familiar, few women had previously heard about PEP or understood how it differed. This may have been because, although PEP is to be taken after a potential HIV exposure, most WESW described extended periods of risk and ongoing HIV prevention needs:

*“I don’t know the difference between PrEP and PEP. I am taking one of them– I have used one which you take after risk exposure (PEP) but I don’t differentiate them with the ones we continuously take before getting exposed (PrEP).”***WESW FGD #1**,** Participant #5**.

WESW also described misconceptions about oral PrEP and PEP being the same as HIV treatment. Women raised concerns that confusion between pills for HIV prevention and treatment could also cause rumors about one’s HIV status, which could have detrimental effects on one’s sex work business:

*“There are some who don’t not know about PrEP and they see you taking the pills they [say] you are HIV positive. In most cases*,* we take PrEP in hiding to avoid rumor mongering by fellow sex workers that you are HIV positive which may affect our work especially when…some of them use it to be able to take way our customers.”***WESW FGD #1**,** Participant #9**.

WESW, peer mentors, and other stakeholders identified opportunities for a peer-mentor led oral PrEP and PEP program to address these capability determinants. First, community-level PrEP and PEP demand creation activities for WESW, led by peer mentors, could increase knowledge about PrEP and PEP and reduce misconceptions. Hearing about a peer’s PrEP and PEP use can facilitate knowledge sharing and encourage others to initiate:

“There were some health workers who came at our hot spot and taught me about PrEP and how it helps prevent one from getting infected with HIV. After the discussion, I initiated on PrEP and after I shared about the same with fellow sex workers at our hot spot. [They] started taking it too.” **Peer Mentor FGD #2**,** Participant #3.**

Second, after WESW initiate PrEP or PEP, peer mentors could provide counseling on adherence, side effects, and stopping and restarting by speaking from their own experiences. Third, they can build ongoing relationships with WESW in their own communities which may enable them to answer questions about oral PrEP and PEP use as they arise and provide dosing reminders. Finally, peer mentors can be a link between the clinics and WESW, by learning about PrEP and PEP from health care workers and bringing that knowledge back to their community. The peer mentor can also bring knowledge about WESW needs back to the health care workers to improve ongoing clinic-based service delivery for this population:

“The peer can [make it] easy for [WESW] to get information from the community and will also inform us (health care workers) about it. As health workers…it will be easy to communicate to the peer mentors who can easily identify the [WESW] who are supposed to get the drugs…They will also help to support in creating awareness.” **Health care worker IDI**,** Participant #5**.

Enabling peer mentors to increase community knowledge and understanding of PrEP/PEP will also require addressing their capability with education about what PrEP and PEP are and how they can be taken.

### Opportunity: Flexible, Community-Based Oral PrEP and PEP Refill and Counseling Options

The social and physical environment created barriers to the opportunity to take PrEP and PEP. Socially, perceived and experienced community stigma around oral PrEP and PEP prevented some WESW from wanting to initiate PrEP/PEP or to be seen with pills: *“Others fear to go to clinic to pick up [PrEP] because they don’t want to meet people who will think that they are HIV positive”* (**WESW FGD #3**,** Participant #10**). Their physical environment where they engage in sex work was also described as a barrier because WESW would sometimes have difficulties packing oral PrEP when relocating or storing pills in locations where they could remember to take them but where they would not be discovered by clients.

To date, WESW in Uganda have generally received oral PrEP and PEP from health facilities or from health care workers conducting community outreach. However, participants described long clinic wait times and lines and PrEP and PEP medication stock-outs that prevented them from accessing HIV prevention options. Clinic operating hours also generally do not align with times when they are available to attend. Several WESW also described lack of transport money to reach the clinic as another barrier. Although outreach visits from the health care workers to the community have been helpful for initiating some women on PrEP and PEP, WESW said that these workers sometimes do not return to the outreach sites with medication refills.

To address these opportunity-related barriers to oral PrEP and PEP use for WESW, participants described a number of potential approaches to move HIV prevention services into the community in a flexible, client-centered way. First, they suggested offering PrEP and PEP at several different community locations, including bars, lodges, and the homes of peer mentors:

“They will like it because it will be convenient since they won’t be walking long distances looking for PrEP or coming to the clinic because it would consume their time. Some would miss their customers while away from their hotspots when they would come for PrEP.” **Peer mentor FGD #1**,** Participant #3**.

Second, a phone or SMS hotline service between WESW and peer mentors could be useful as a way for PrEP and PEP clients to report when refills are needed or to ask about any medication challenges. Flexible oral PrEP refill options (e.g. 3-month or longer refills) would be useful for WESW who have different abilities to store pills. Finally, peer mentor-led oral PrEP and PEP counseling could help to address some of the concerns around HIV-related stigma while providing regular support where WESW are living and working:

“The greatest benefit will be that this community that you are reaching out to will have someone who is like a point of reference when it comes to PrEP and PEP. That is very important because our [health care] facilities are still very few…meaning that most of the people cannot easily access the services. I think this is an opportunity to build the capacity of the community members to support sensitization and engagement in HIV services.” **Key Informant IDI**,** Participant #3**.

### Motivation: Empowering, Integrated, and Safe Spaces for Oral PrEP and PEP Delivery

In contrast with findings around capability and opportunity to use PrEP and PEP, conversations around PrEP and PEP motivations were largely from a positive lens of discussing facilitators of medication use. WESW described reflective motivations to take oral PrEP and PEP related to how their medication use would align with their goals and intentions to live an HIV-free life. Specifically, women discussed their desires to prevent HIV acquisition and stay healthy for their own futures and for the future of their children: *“I realized I needed [PrEP] because if the nature of the job that I am doing – I need good health to be able to bring up my children”* (**WESW FGD #1**,** Participant #3**). They also talked about the importance of remaining healthy to continue engaging in sex work as an economic opportunity. These goals aligned with feelings of optimism about the HIV prevention benefits of PrEP and PEP, which were reinforced by hearing positive stories from WESW peers who have remained HIV negative: *“I have seen people testifying that they have used PrEP for a couple of years and are still negative and that motivated me”* (**WESW FGD #1**,** Participant #10**).

Motivations to take PEP differed slightly from motivations to take oral PrEP. WESW described PEP as a short-term HIV prevention option that would be useful around instances of sexual violence, when clients refused to use condoms, or after forgetting to take their PrEP: *“If things go bad and a man refuses to wear a condom*,* we can go get PEP pills and take them”* (**WESW FGD #1**,** Participant #9**). Daily oral PrEP was seen as a longer-term prevention option that could protect from HIV throughout the day-to-day continual HIV exposure that comes from engaging in sex work.

In suggesting intervention components for a peer mentor PrEP and PEP delivery approach, FGD and IDI participants identified ways to bolster and capitalize on these facilitating motivations to reinforce their influence on PrEP and PEP use for WESW. For example, all participants described the importance of conducting a rigorous selection process to identify peer mentors to ensure that they are trusted members of the community who can speak to their own PrEP and PEP experiences in a positive way that will encourage other WESW through their perspectives:

“There should be a chance of giving testimonies about PrEP by those already using it and this will motivate others too. For instance, I have been on PrEP for some years while engaging in sex work with different men and I am still HIV negative. We should come out and share such information…it will motivate [WESW] to initiate and, those who are already on it, to adhere.” **Peer Mentor FGD #2**,** Participant #7**.

Given that PrEP and PEP desires were grounded in a hope of remaining healthy for oneself and one’s children, participants also discussed an integrated service delivery approach that could combine HIV prevention medications with reproductive health services and other basic health care. Finally, peer mentors could offer WESW HIV self-testing kits along with oral PrEP and PEP, which would create opportunities to reinforce PrEP and PEP motivations with discussions about negative HIV test results. Specifically, WESW liked that HIV self-testing provides discreet information on one’s HIV status (*“I have liked the HIV self-testing kits because I may not like another person to know my HIV test results”*, **WESW FGD #1**,** Participant #3**) and results could provide reassurance about the continued need for PrEP and PEP.

## Discussion

In this formative study among WESW, peer mentors, and stakeholders in southwestern Uganda, participants expressed high interest in a community-based, peer-led intervention to address barriers to oral PrEP and PEP use among WESW. Participants described barriers to oral PrEP and PEP access and adherence across the three COM-B constructs: capability to know about these biomedical prevention tools, opportunity to receive, store, and take them, and motivation to prevent HIV as a driver of use. Discussions of these barriers led to the identification of possible components of a peer-led oral PrEP and PEP delivery strategy, including PrEP demand creation from peer mentors, PrEP and PEP delivery in convenient community locations, phone or SMS hotline services, flexible PrEP refill options, and HIV self-test kits. Our work is one of the first in-depth inquiries into peer-led approaches for providing both oral PrEP and PEP in community settings for WESW. With global oral PrEP and PEP guidelines now recommending decentralized HIV prevention services in community spaces [28–34], novel community-based, peer-led PrEP and PEP delivery models merit further testing.

Our findings highlight opportunities to build on momentum for decentralized, demedicalized HIV prevention service delivery in a setting where peer approaches are already endorsed in health guidelines. Peer approaches have been successfully deployed in other settings and key populations for oral PrEP delivery, including in Uganda and Thailand [35, 36]. Peer services have also been used for HIV prevention efforts among WESW in Southern Africa [37], but peers have not been widely involved in the actual delivery of biomedical HIV prevention options like oral PrEP and PEP in community settings. A recent pilot study of peer-delivered HIV self-tests and daily oral PrEP among young women in Uganda found that this approach was acceptable and feasible but that gaps remained in offering ongoing peer support around oral PrEP refills [38].

Our findings build on prior research on peer approaches for HIV prevention and suggest that peers could play a key role in addressing barriers to oral PrEP and PEP use among WESW. Capability-related barriers, such as misconceptions about PrEP and PEP and limited knowledge of PEP, could be addressed with community-based PrEP demand creation led by peer mentors, PrEP and PEP counseling from peers who can provide support based on their own personal experiences, and relationship building between WESW, peer mentors, and health facility staff. Opportunity-related barriers, including community stigma, inability to store oral PrEP pills, and facility lines could be addressed with peer-led, community-based PrEP delivery in convenient locations and flexible models for refills and phone-based support. Motivation-related barriers, such as aligning oral PrEP use with one’s intrinsic HIV prevention, could be mitigated with HIV self-test kits to empower knowledge of HIV status, integrated health care delivery to holistically address overall health and wellbeing needs, and the selection of trusted and respected peer mentors.

Our findings add to the literature on barriers to PEP use for WESW. While our study participants described interest in PEP and the key distinctions between PEP and PrEP that would influence their choice (e.g., duration of potential HIV exposure), WESW in Uganda and other settings generally describe low knowledge and awareness of PEP and few have experience using it [30, 39–41]. Given recent updates to WHO PEP guidelines in 2024 to improve uptake of and adherence to PEP globally [34], this work provides timely evidence on current barriers to PEP use among WESW in Uganda. The barriers to oral PrEP uptake and adherence identified in our work are similar to those reported in several other studies with WESW. For example, prior research with WESW in South Africa, Uganda, and Zimbabwe found that stigma and structural barriers (e.g., transportation challenges, costs) are barriers to PrEP uptake and adherence [14–16, 42–44].

This work offers a case study in the use of a theoretical framework, the COM-B, to identify barriers to PrEP and PEP use and possible implementation approaches to address these determinants via a peer-led delivery model. The COM-B model is an evidence-based approach to organize determinants of behavior change to inform intervention development [26]. It has been used to develop multi-component interventions for PrEP adherence among adolescent girls in the United States [45], tuberculosis contact identification in Uganda [46], and hypertension care among HIV-affected adults in Uganda [47]. We used the COM-B model to link determinants to potential elements of a peer-led oral PrEP and PEP delivery intervention. Our qualitative findings inform a novel peer-led PrEP and PEP delivery intervention for WESW and, in a subsequent trial, we will test whether the intervention successfully increases use of biomedical HIV prevention through the determinants identified using our COM-B model. This work provides a model of theory-informed intervention development for peer-led HIV prevention services that could potentially be expanded upon as long-acting prevention options become available in Uganda to advance an agenda of differentiated, person-centered HIV prevention among key populations [28]. However, the COM-B model is primarily focused on determinants of individual-level behavior change (PrEP/PEP uptake or adherence among WESW, PrEP/PEP delivery challenges among peer mentors) and does not comprehensively explore contextual, structural, and institutional-level factors that might drive PrEP and PEP implementation. While we were able to learn about some training and clinic and community needs from peer mentors and key informants, evaluation of our peer-led PrEP and PEP delivery model must also consider cost, health disparities and social and structural determinants of health among WESW in Uganda, and PrEP and PEP regulatory guidelines for community versus clinic distribution [48].

While our work is unique for its focus on WESW and inclusion of both oral PrEP and PEP, several of the identified peer-led components have been acceptably and feasibly included in prior HIV programs for young women in Southern and Eastern Africa. For example, the DREAMS HIV prevention program in Uganda offered oral PrEP adherence support via peer-delivered phone communication to young women, who reported high acceptability of the service [49]. Young mothers living with HIV in Malawi, Tanzania, Uganda, and Zambia appreciated receiving ART adherence supportive counseling from other young mothers, echoing the importance of receiving counseling from trusted peers going through similar journeys [50]. Recent pilot studies in Uganda offering HIV self-testing and oral PrEP to adolescent girls and transgender women via peers found that peer-delivery was associated with high PrEP uptake and adherence and participants found it motivating to receive oral PrEP from a “non-judgmental” person in their community [38, 51]. One study of integrated daily oral PrEP and sexual and reproductive health services, including STI testing and treatment, with peer navigators for girls in South Africa found high linkage to PrEP [52]. While our work adds to this literature on peer delivery models specifically for oral PrEP (which was the only biomedical HIV prevention option publicly available in Uganda at the time of this study), additional considerations will likely be needed to adapt peer-led HIV prevention models to include long-acting PrEP products like cabotegravir and lenacapavir injections. For example, when long-acting PrEP options become available in Uganda, PrEP education will need to include discussion of tradeoffs and choice around various prevention products. Peer mentors would also need to manage different requirements around HIV testing and follow-up visit cadence in accordance with product dosage. Future research will be needed to adapt peer-led PrEP delivery models to new, longer-acting options as product choices expand.

Participants highlighted the need to select trusted peer mentors and provide adequate training and supervision to ensure the success of peer-led PrEP and PEP delivery. Because peer mentors are likely to be other WESW with similar experiences to their clients, it will be important to help them manage any ongoing stressors that may arise in hearing the stories of other WESW and also in clearly defining the scope of their roles [53]. Peer mentors must be scaffolded by qualified supervisors who can provide ongoing training and supervision in counseling approaches, PrEP and PEP knowledge, and confidential communication and will also need the buy-in and support of providers at local healthcare clinics [53].

The strengths of this study include a broad sample including WESW who would be end-users of our future intervention and individuals with experience working in a peer mentor role in Uganda. We conducted a mix of KIIs and FGDs, which enabled us to learn nuanced information on personal experiences and barriers to oral PrEP and PEP use and to hold group brainstorming discussions on peer-led solutions to barriers. Our work was grounded in the COM-B model, which enhances the rigor and comparability of our findings. This work also fills a gap in the literature on use of PEP among WESW. This study had a number of limitations. Only a subset of our IDI participants had prior experience with using daily oral PrEP and/or PEP before data collection and we did not collect survey data among all participants to systematically measure prior PrEP and/or PEP use experience. For PrEP and PEP naïve participants, discussions around barriers to PrEP and PEP use were based on issues accessing these products and hypothetical issues that may arise if they were offered these products. Based on our prior work offering oral PrEP and PEP in this setting [30, 32, 33, 54], we previously hypothesized that peer-delivered HIV prevention services would be acceptable for WESW and offered a number of example peer-led intervention components a priori during the KIIs and FGDs (e.g., phone/SMS hotline to request refills and report challenges, flexible PrEP refill options, HIV self testing). While these examples catalyzed discussions around peer-led delivery strategies, they also may have biased responses. However, KII and FGD facilitators were trained in techniques to mitigate bias (e.g., avoiding asking leading or closed-ended questions) and we included open-ended questions about other ideas for intervention components to address barriers to PrEP and PEP use. This research was conducted in peri-urban communities in Uganda and our findings may not be generalizable to WESW in more urban contexts, those with greater higher density of clinics and greater community knowledge of PrEP and PEP, and in countries where sex work is permitted.

## Conclusions

We identified barriers to oral PrEP and PEP access and adherence among WESW in rural Uganda including issues around capability, opportunity, and motivation to use these prevention medications. Based on these barriers, we then identified several key components of a peer mentor-led PrEP and PEP delivery model that could enhance HIV prevention product access and adherence in this population. This intervention should now be tested to determine its impact on oral PrEP and PEP use.

This work is timely given that community-based oral PrEP and PEP delivery is being scaled up and peer-led HIV service delivery models are being explored in Uganda and elsewhere [28, 52–58]. Future community-oriented oral PrEP and PEP programs could consider including the components identified here to facilitate peer-led service delivery, which has the potential to increase PrEP and PEP knowledge, reduce stigma around HIV services, and improve person-centered care with the ultimate goals of expanding the reach and effectiveness of HIV prevention services among WESW.

## Data Availability

De-identified debrief report, memo, and/or matrix data available upon request.
